# X-ray Photoelectron Spectroscopic Study of Some Organic and Inorganic Modified Clay Minerals †

**DOI:** 10.3390/ma14237115

**Published:** 2021-11-23

**Authors:** J. Theo Kloprogge, Concepcion P. Ponce, Danilo O. Ortillo

**Affiliations:** 1 School of Earth and Environmental Sciences, The University of Queensland, Brisbane, QLD 4072, Australia; j.kloprogge@uq.edu.au; 2 Department of Chemistry, College of Arts and Sciences, University of the Philippines Visayas, Miagao, Iloilo 5023, Philippines; cpponce@upv.edu.ph

**Keywords:** X-ray photoelectron spectroscopy, clay intercalation, pillared clays, kaolinite, montmorillonite, hydrotalcite

## Abstract

Layered clay systems intercalated with inorganic and organic compounds were analyzed to highlight how XPS can provide information on the different environments surrounding a particular atom as well as provide discernments on the size, coordination, and structural and oxidative transformations of the intercalating/pillaring compounds. XPS data on the intercalation of urea and K-acetate in low- and high-defect kaolinite revealed the interaction of the intercalating group NH_2_ with the siloxane functional groups in the interlayer surface. The intercalation of HDTMA in Mt demonstrated the use of XPS in monitoring the change in conformation assumed by alkylammonium intercalating compounds in Mt with increasing CEC. Studies on the pillaring of Mt by Al_13_ and Ga_13_ by XPS allowed determination of the coordination of the pillaring compound within the Mt layer. Lastly, the intercalation of hexacyanoferrate in hydrotalcite demonstrated the capability of XPS in following changes in the oxidation state of the iron compound. These were gleaned from interpretation of the shifts in binding energies and presence of multiplet splitting in the XPS of the component elements of the minerals or the intercalating compounds.

## 1. Introduction

Clay minerals can host a large number of functional organic and inorganic systems through a process called intercalation. These intercalated systems can impart new and/or unique properties that afford wide ranging applications of these modified clay minerals in various fields such as in environmental remediation technologies [1]; cosmetics and pharmaceutical science [2]; material science [3]; carbon dioxide capture and storage [4]; and abiogenesis [5] to name a few. The driving forces of the intercalation process includes intermolecular forces of interactions between the clay mineral interlayer surfaces and the intercalating molecule (e.g., hydrogen-bonding, ion-dipole interactions, and van der Waals attractions) [6]. The formation of coordinate bonding can also drive intercalation as in the case with aniline intercalated in Ca^2+^, Cu^2+^, Fe^3+^-exchanged montmorillonite [7]. In some instances, the acid-base behavior of clay interlayer surfaces or the intercalating compound drives the intercalation process [8,9]. Understanding and control of the above-mentioned interactions of the clay minerals with the intercalating or with the pillaring compound relies on the researchers’ abilities to use innovative approaches in probing these interactions.

Physicochemical characterizations of different types of intercalated and pillared clays have been carried out using various spectroscopic techniques to monitor insertion or intercalation of specific compounds into raw clays. Raman and FTIR techniques are widely available to probe the structure and structural changes in intercalated and pillared clay minerals through monitoring the characteristic vibrational frequencies of functional groups present and the changes they undergo [10,11]. For example, FTIR, in conjunction with data on water vapor pressure dependence of *d*-spacing in Na- and Ca-montmorillonite (Mt), was used to distinguish various hydration states of the Mt mineral [12]. The validation of the use of FTIR vibrational frequencies to distinguish various hydrogen bonding environments in dry (0 W), 1 W, and 2 W interlayers in Mt provides a facile and accessible technique to understand the geochemical and technological reactions of water film-bearing Mt. Mid-IR and IR techniques were also used to probe the structural and surface properties of Mt modified with organic compounds [11,13]. Slaný, Jankovič, and Madejová intercalated in Na-Mt a series of primary alkylamines (C1-C19) and their ammonium salts [11]. Their results, especially those of the near-IR (NIR) vibrational frequencies, show that the head-group structure (e.g., NH_2_ or NH_3_^+^) as well as the counterpart anions (chloride or the Mt layer) influenced the shape and position of the N-H vibrational frequencies as a consequence of the different H-bonding natures in the compounds studied [11]. They also showed that NIR vibrational frequencies are sensitive to the conformational changes of the alkylchains in the Mt layer and were able to distinguish groups differing in the trans/gauche conformer ratio [11]. Another technique used to study structural changes in clay minerals is X-ray diffraction (XRD). The technique is used to semi-quantitatively determine mineral content and in particular changes in basal spacing, (d_(001)_), which is a measure of the size and orientation of the organic molecule or of the inorganic pillar in the interlayer space [14,15]. Changes in d_(001)_ of layered clays are often used to confirm successful intercalation of organic compounds [11,16,17,18]. Basal spacing values were also employed to conjecture that long chain alkylammonium ions were packed as monolayer, bilayer, pseudotrimolecular layer, or paraffin-type monolayer within 2:1 clay minerals [15]. While vibrational frequencies and X-ray diffraction techniques are undoubtedly efficient and accessible techniques to probe the intercalation of inorganic and organic molecules in clays, x-ray photoelectron spectroscopy (XPS), also known as electron spectroscopy for chemical analysis (ESCA), is a complementary technique that can be used to determine the chemical composition of these modified clay materials [19,20,21,22,23,24,25,26]. Though generally considered a surface analysis technique, more than 90% of the signal comes from the bulk of the sample analyzed, hence it makes it a strong potential analytical tool for the analysis of clay minerals that are generally in the nanometer size range. High energy X-rays applied to the samples can initiate core excitations that can provide information about the structure and electronic properties of transition in the intercalated and pillared clay catalysts. The technique had been applied to study the effects of an element’s oxidation state, type of bonding, coordination number, and nearest neighbors on its binding energies [22]. Several examples of these applications are enumerated below. Schampera et al. [25] presented the use of XPS to study the intercalation of hexadecylpyridinium cation on bentonite clays and concluded that at small surfactant concentrations, the organic molecules are arranged as monolayers with the alkyl chains parallel to the clay surface, whereas at higher surfactant concentrations the organic molecules are more disorderly with some alkyl chains assuming a tilted orientation relative to the surface. Raeburn, Ilton, and Veblen [27] used the technique along with factor analysis to quantitatively analyze the Fe(III)/ΣFe of biotite single crystals by monitoring the Fe 3p photoelectron peak. XPS was also used to systematically determine the average oxidation states of manganese in birnessites, layered minerals which have applications as catalysts in biomemitic water-oxidation processes [28]. Ilton et al., who conducted this study, have shown that: (1) Mn 3p and Mn 3s lines are accurate in determining the oxidation state regardless of Mn bonding environment of Mn; (2) the Mn 2p3/2 line is useful when fit parameters are fine tuned to the mineral/compound under study to address the sensitivity of the line to the Mn bonding environment; and (3) the accuracy of the Mn 3s multiplet splitting is questionable for birnessite contrary to its common use in establishing oxidation sates of Mn in monovalent oxides [28]. The effect of nearest neighbors on binding energies has been explored by Gonzalez-Elipe et al. [29] by measuring the photoelectron binding energies of Si, O, Al, and Mg natural phyllosilicates with various degrees of isomorphic substitution in either the octahedral or tetrahedral layers. When such substitutions in the tetrahedral sheet result to an excess negative charge, the binding energies of Al_T_, Si, and O decrease in a linear fashion [29]. These few examples demonstrate that the local information provided by XPS cannot always be obtained by other techniques, such as Raman and infrared spectroscopy. This information may manifest as shifts in binding energy or as multiplet splitting due to slightly different environments around a particular atom. Despite providing researchers with a unique tool to directly study the chemistry at the surface of minerals, monitor interfacial interactions, and allow for a better understanding of the local structures and changes in the mineral structures upon intercalation and pillaring, there are limited papers focusing on its applications beyond confirming that intercalation/pillaring occurred.

In this study, XPS studies of selected 2-dimensional clay model systems are showcased: urea and K-acetate intercalated kaolinite [30,31,32,33], hexadecyltrimethylammonium (HDTMA) intercalated montmorillonite [34,35,36], Al_13_ and Ga_13_-pillared montmorillonite, and hexacyanoferrate intercalated hydrotalcite as an example of a so-called intercalated anionic clay [37,38,39,40,41,42]. These are all widely studied and are well suited to highlight how XPS reveal subtleties in the environments around the intercalating organic molecules or the inorganic pillaring agents since data on the widely used Raman, IR, and XRD techniques are readily available for comparison.

## 2. Materials and Methods

### 2.1. Urea and K-Acetate Intercalated Kaolinite

All chemicals used in this study were analytical grade unless otherwise stated. A low defect and a high defect kaolinite sample from Hungary, specifically from Kiralyhegy and Szeg, respectively, are reported in this paper. The urea intercalated kaolinite samples were prepared by mixing 300 mg kaolinite with a 9.0 M urea aqueous solution at 65°C for 80 h. Following the heating step, the excess solution was decanted and the intercalated was kaolinite removed from the remaining solution by centrifugation [32].

The potassium-acetate intercalated kaolinite was prepared by mixing 300 milligrams of kaolinite with 30 mL of 7.2 M saturated K-acetate solution and shaking for 80 h in a constant temperature bath at ambient temperature and separated from the excess solution as described above [43].

### 2.2. Hexadecyltrimethylammonium (HDTMA) Intercalated Montmorillonite

A montmorillonite (Mt) sample from Hebei province, China, was purified by sedimentation. This particular Mt sample has the general formula [Na_0.05_Ca_0.18_Mg_0.10_][Al_1.58_Fe_0.03_Mg_0.39_][Si_3.77_Al_0.23_]O_10_(OH)_2_·nH_2_O. Only the <2-μm fraction was collected and dried at 90 °C. The Mt was ground and sieved through 200 mesh and sealed in a glass tube for use. This Mt has a 57.9 meq/100 g cation exchange capacity (CEC). Hexadecyltrimethylammonium bromide (HDTMABr) with a purity of 99% from YuanJu Chem. Co., China, was used without any further purification as the surfactant to intercalate the Mt with. First, an Na-exchanged montmorillonite (Na–Mt) was prepared by mixing 9.4 g Mt and 0.6 g Na_2_CO_3_ in 100 mL of deionized water and stirring at 80 °C for 3 h. Then, the resulting Na–Mt was separated by centrifugation and washed with deionized water until the pH of the solution was 7. The Na–Mt was dried at 105 °C, ground and sieved through 200 mesh and stored in a sealed bottle. The intercalation of the Mt was performed by firstly suspending 2.5 g of Na–Mt in about 300 mL of deionized water followed by the slow addition of the desired amount of HDTMABr. The concentrations of HDTMA^+^ used were equivalent to 0.5 CEC, 0.7 CEC, 1.0 CEC, 1.5 CEC, 2.0 CEC, and 2.5 CEC of Mt, respectively. Samples are labeled as 0.5CEC-Mt, 0.7CEC-Mt, etc. The reaction mixtures were stirred for 10 h at 80 °C. All intercalated Mt were washed free of Br^−^ anions (tested by AgNO_3_) using deionized water, dried at 90 °C, and ground in an agate mortar [34].

### 2.3. Pillared Clays

Na–Mt SWy-2 from the Source Clay Repository of the Clay Mineral Society was subjected to gravity sedimentation and decantation to obtain < 2 μm fractions then washed 4 times with 1 M NaCl solution. The excess electrolyte was then removed by washing five times with deionized water. Meanwhile, aluminum tridecamer, Al_13_ [(AlO_4_Al_12_(OH)_24_(H_2_O)_12_)^7+^], solutions were prepared as follows: 0.2 M Na OH (Merck KGaA, Darmstadt, Germany) solution was injected at a rate of 0.015 mL/s using a Gilson pump (capillary diameter of 0.5 mm) below the surface of an 0.5 M Al(NO_3_)_3_ (Merck KGaA, Darmstadt, Germany) solution, while vigorously stirring until the OH/Al molar ratio was 2.4. These solutions, which comprised approximately 70% Al_13_ polymer [44], were then rapidly added under vigorous stirring to specified volumes of clay suspensions containing 20 g of solid/l to attain ratios of 5.5 and 20 meq Al/ g clay. Ion exchange was continued for 12 h after which the intercalated clays were washed five times with deionized water, followed by air- or freeze-drying and calcination at 973 K to obtain the final pillared clay [42].

The preparation of Al_12_Ga-pillared clay and Ga_13_-pillared clay employed Miles Mt (Miles, Queensland, Australia) and followed a procedure analogous to that of the Al_13_-pillared clay. First, a 0.1 M solution of NaOH was pumped peristaltically at a rate of 0.01 mL/min to a vigorously stirred Ga(NO_3_)_3_ (Merck, KGaA, Darmstadt, Germany) at ambient temperature. The ratio of OH/Ga in the prepared solution was 2:1. The Al_12_Ga and Ga_13_ solutions were then added to the aqueous Mt suspensions under continuous stirring for 4 h and then allowed to stand for 5 days. The intercalated clays were washed 5 times with deionized water and separated using a centrifuge. The solids were subsequently dried in air at ambient temperature. Finally, the samples were heated at 2 K/min and calcined at 723 K for 8 h to obtain the final pillared clay [39].

### 2.4. Hexacyanoferrate Intercalated Hydrotalcite

Hexacyanoferrate intercalated hydrotalcite was prepared following the procedure described by Frost et al. [37]. Briefly, aluminum and magnesium nitrates were dissolved in freshly decarbonated water to obtain a final concentration of [Al^3+^] = 0.25 M and [Mg^2+^] = 0.75 M. The mixed solution was purged with N_2_ for 20 min. In another vessel, a mixture of sodium hydroxide ([OH^−^] = 2 M) and the desired anion (hexacyanoferrate(II), [Fe(CN)_6_]^4−^, and hexacyanoferrate(III), [Fe(CN)_6_]^3−^), at the appropriate concentration were prepared and concurrently purged with N_2_ for 20 min as the cationic mixture (all compounds used were obtained from Merck KGaA, Darmstadt, Germany). The cationic solution was pumped peristaltically to the anions at 40 mL/min. The pH of the mixture was maintained above 9 with the addition of 0.1M NaOH. The precipitate was then aged at 75 °C for 18 h under a N_2_ atmosphere and subsequently washed thoroughly with ambient temperature decarbonated water to dissolve remaining nitrates and dried in a vacuum desiccator for several days [37].

### 2.5. X-ray Photoelectron Spectroscopy (XPS)

The XPS spectra were collected using a Kratos AXIS Ultra (Kratos Analytical, Manchester, U.K.) equipped with a monochromatic 225 W Al X-ray source under ultrahigh vacuum conditions. Preliminary scans were performed with instrument set at 0 to 1200 eV survey scan, dwell time of 100 milliseconds, pass energy of 160 eV, and step size of 1 eV with 1 sweep. High resolution analyses were then performed by increasing the number of sweeps, lowering the pass energy to 20 eV at steps of 100 meV and increasing the dwell time to 250 milliseconds. The adventitious C 1s C-C peak at 284.8 eV was used to charge correct all spectra. 

## 3. Results and Discussion

### 3.1. Clays Intercalated with Organic Molecules

Kaolinite has frequently been depicted as a clay mineral that has a low capacity to expand, contrary to the swellable 2:1 clay minerals—smectites, which include montmorillonite, saponite, etc. Its crystal structure can be described as a repetition of layers comprising a single Al(O_2_OH) octahedral sheet attached to a single SiO_4_ tetrahedral sheet. Within this layer structure, four different OH-groups in the octahedral sheet are present. Three of these OH-groups point out of the layer’s octahedral sheet and form hydrogen bonds with the next layer’s siloxane surface of the tetrahedral sheet. These OH-groups are called the inner surface (or on occasion called outer) OH-groups. The last OH-group is oriented into the free space between the tetrahedral and octahedral sheets and is called the inner OH-group. Currently, various organic molecules such as urea (NH_2_-C=O-NH_2_) and potassium acetate (K-CH_3_CO_2_) have been identified with suitable proportions and bonding characteristics that can be intercalated in the kaolinite interlayer space. Intercalation of these molecules can occur through various mechanisms contingent upon the locations of the interactions between the organic molecule and the kaolinite interlayer surfaces. The reactive organic guest molecule penetrates into the interlayer space between adjacent kaolinite layers. This disrupts the hydrogen bonds between the inner-surface OH-groups of the Al octahedral sheet and the oxygen atoms of the Si tetrahedral sheet. The intercalated organic molecule can then interact through hydrogen bonding with the more hydrophobic siloxane surface and/or with the more hydrophilic OH-groups of the octahedral sheet. Much of these interactions in intercalated structures have been elucidated by mid-infrared and Raman spectroscopy (see refs. [11,33,45,46,47]), but XPS can potentially provide additional information.

#### 3.1.1. Urea Intercalated Kaolinite

Urea, CO(NH_2_)_2_, is an amide with two –NH_2_ groups joined by a carbonyl functional group. In its pure form, urea is characterized by a single C 1s peak and a single N 1s peak as both NH_2_ groups are identical. For poly(urea) the C=O C 1 s peak has been observed at binding energy (BE) = 288.8 eV and the N 1s at 399.9 eV [48].

**Figure 1:**
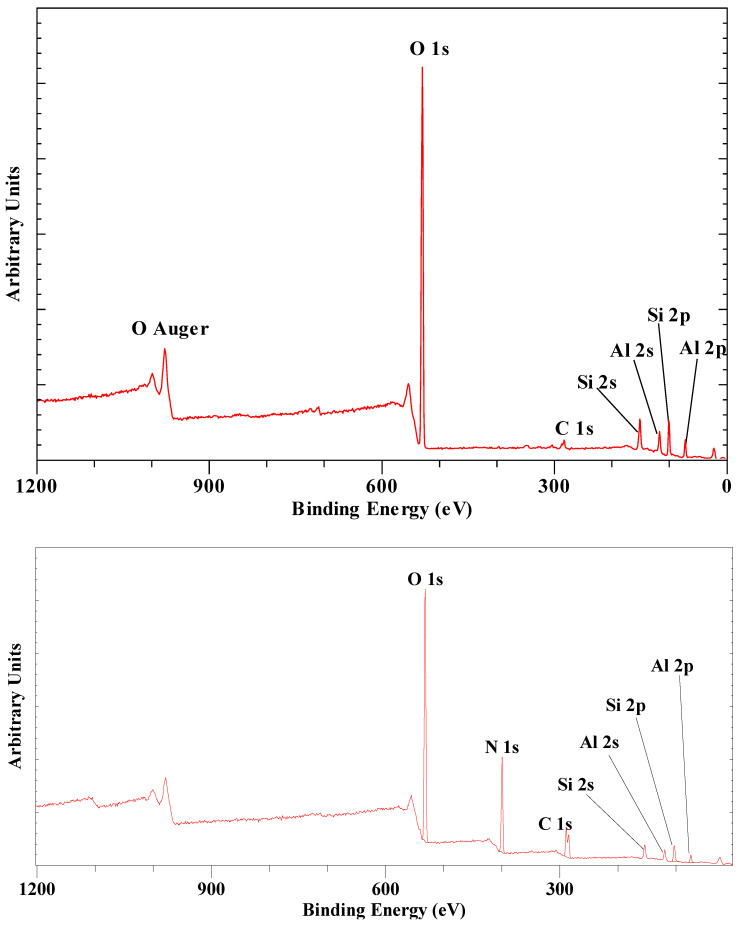
XPS survey scan between 1200 and 0 eV of (**top**) Szeg kaolinite and (**bottom**) urea intercalated Szeg kaolinite showing beside the Al and Si peaks of the kaolinite a complex C 1s and N 1S of the intercalated urea in the bottom figure.

**Figure 2:**
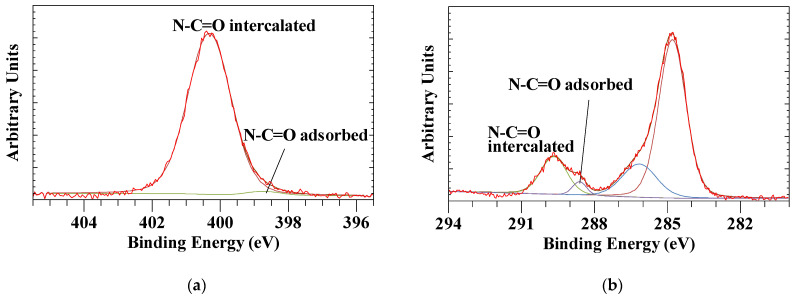
XPS high resolution spectra of intercalated Szeg kaolinite (**a**) N 1s and (**b**) C 1s showing both intercalated and adsorbed urea peaks. The XPS high resolution spectra of Kiralyhegy kaolinite show similar features and are not shown here.

The XPS survey scan of urea intercalated Szeg kaolinite is characterized by the Al and Si peaks associated with the kaolinite structure in addition to a complex C 1s and N 1s of the intercalated urea (Figure 1). Only a single C 1s peak and no N 1s peak was observed for pure kaolinite [47,49]. The N 1s high resolution spectrum of the intercalated urea is characterized by a strong single peak at BE = 400.31 eV with a minor peak at 398.54 eV which is probably related to a small amount of urea adsorbed on the Szeg kaolinite crystal’s external surface (Figure 2a). In comparison, the intercalated N 1s peak for the Kiralyhegy kaolinite was observed at a slightly lower binding energy of 399.93 eV. The C 1s high resolution spectrum consists of several peaks (Figure 2b). The peak at 289.67 eV is associated with the intercalated urea, while a second peak at a slightly lower binding energy of 288.61 eV is probably related to the urea adsorbed on the external surface. The peaks at ca. 284.8 and 286.3 are due to adventitious C 1s C-C peak present in all surfaces. For the Kiralyhegy kaolinite, the urea C 1s peaks were observed at 0.1 eV higher binding energies. The kaolinite layer structure shows minor effects because of the intercalation. The Al 2p3/2 shifts from 74.53 eV to 74.67 eV which is basically within the experimental error of the XPS instrument. In contrast, the Si 2p3/2 shows a larger shift upon intercalation from 102.76 eV to 100.34 eV. Interestingly, this is opposite to what one would expect based on earlier infrared and Raman spectroscopic results which indicates a hydrogen bond of the C=O with the OH-groups of the octahedral sheet where the Al is located and there is only minor interaction with the siloxane surface [50].

Ledoux and White [12] have used IR techniques in probing the interactions of intercalated urea with functional groups in kaolinite. They have suggested that urea interacted with inner-surface OH-groups. They studied the possible orientations of the transition moments in intercalated urea molecules by looking at the IR pleochroism of urea intercalated kaolinite after heating at 110 °C for 5 h. A one-dimensional Fourier-projection of the urea intercalated kaolinite indicated an alignment of the intercalated urea molecules with one NH_2_ group located near the siloxane surface O atoms and the remaining NH_2_ and CO groups were in the vicinity of the inner-surface OH-groups. As a result, the urea intercalated in kaolinite now has NH_2_ groups in different environments.

The splitting of the Raman band at 3620 cm^−1^ (assigned to the inner OH-stretching vibration) into two separate vibrations at 3615 and 3621 cm^−1,^ which was observed when Frost et al. [1] cooled urea-intercalated kaolinite to −196 °C, strongly indicated two different types of interactions of urea. One is the formation of hydrogen bonds between the C=O group and the inner surface OH-groups and another is between the NH_2_ groups with the tetrahedral sheet’s siloxane surface; the other NH_2_ group remained free, supporting the observations by Ledoux and White [12]. It is also possible that the vibration at 3620 cm^−1^ at ambient temperature was a result of a strong overlap of the vibrations of the inner OH-group and of the C=O which is hydrogen bonded with the inner-surface OH-group. Another explanation is supported by the observed C-N stretching region, where the vibration at 1009 cm^−1^ at ambient temperature moved to 1014 cm^−1^ together with a broad shoulder at 1004 cm^−1^ upon decreasing the temperature to −196 °C. This is indicative of two different NH_2_ groups in the urea molecule after intercalation. Thus, Frost et al. [1] posited that at ambient temperature, the urea only formed a bond to the siloxane surface. When the temperature was decreased to −196 °C, the C=O group of the urea and the OH-groups moved so close to each other that they were able to form a new hydrogen bond. Based on this information, it can be concluded that the observation of two different environments for the NH_2_ groups in the intercalated kaolinite is not large enough to be observed in the N 1s high resolution spectrum, although the influence of the NH_2_ group on the siloxane layer may explain the shift in the Si 2p3/2 by around 2 eV.

#### 3.1.2. K-Acetate Intercalated Kaolinite

**Figure 3:**
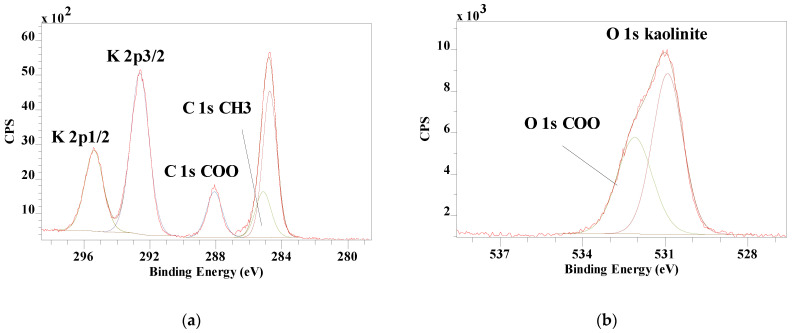
High resolutions XPS spectra of potassium-intercalated Kiralyhegy kaolinite showing (**a**) C 1s and K 2p and (**b**) O 1s.

Potassium acetate (CH_3_COOK), the potassium salt of acetic acid, is a hygroscopic solid at 273 K. XPS analysis of acetic acid adsorbed on ice by Křepelová, Bartels-Rausch, Brown, Bluhm, and Ammann [23] showed the C 1s of the CH_3_ group at 285.5 eV and the COO_-_ group at 289. 3 eV, while the two O 1s peaks were observed at 532.3 and 533.7 eV. Comparable results were published by Kong et al. [51] for sodium acetate, but with only a single O 1s peak for COO^−^ at 531.5 eV. The low defect kaolinite from Kiralyhegy and the high defect kaolinite from Szeg show increased C 1s peaks and the appearance of K 2p and K 2s peaks after intercalation with K-acetate survey scans. For the Kiralyhegy kaolinite, K 2p3/2 was observed at 292.62 eV with the C 1s for CH_3_ at 285.79 eV and COO^−^ at 288.10 eV (Figure 3). The O 1s clearly showed the presence of two peaks at 530.92 eV and 532.11 eV compared with one peak at 531.93 eV for pure Kiralyhegy kaolinite. 

Both the Si 2p3/2 and Al 2p3/2 are slightly shifted (less than 0.2 eV) to lower binding energies but the difference is so small that it is within the experimental error of the XPS instrument. For the Szeg kaolinite, the K 2p3/2 was observed at 292.61 eV with the C 1s for the CH_3_ group at 285.11 eV and for the COO- group at 288.15 eV. The O 1s clearly showed the presence of a strong peak at 530.90 eV and two weak peaks at 532.0.6 and 532.95 eV compared with one peak at 531.78 eV for pure Szeg kaolinite. Both the Si 2p3/2 and Al 2p3/2 exhibited any shift upon intercalation. The difference between high defect (Kiralyhegy) and low defect (Szeg) kaolinite on the intercalation of potassium acetate results in some differences in the binding energies of the elements of the potassium acetate. The K 2p3/2 is not affected by the difference in kaolinite defect structure as well as the C 1s for the carboxylic acid group. The aliphatic group however is showing a difference in binding energy of 0.68 eV. The other difference is observed in the O 1s where there are only two peaks present in the intercalated high defect kaolinite and three peaks in the intercalated low defect kaolinite. The main peak at 530.9 eV is related to the O 1s of the kaolinite structure and is shifted by about 1 eV compared with the pure kaolinites. The low defect kaolinite structure seems to result in a better ordering of the potassium acetate in the interlayer space allowing to observe the two oxygen atoms in the carboxylic acid group separately. 

The intercalation of kaolinite with potassium acetate has been extensively studied by infrared and Raman spectroscopy [50]. Intercalation with potassium acetate produced a new Raman active vibration at 3605 cm^−1^. There was no observable change in intensity in the stretching vibration assigned to the inner OH-, but a substantial broadening was observed relative to the starting kaolinite. It is conceivable that the K cation has the right size to occupy the ditrigonal hole of the siloxane sheet (tetrahedral sheet) of the kaolinite, thus affecting the polarization of the inner OH-groups [52]. The vibrations attributed to the inner-surface OH-groups strongly decreased in intensity after intercalation with potassium acetate pointing to the formation of hydrogen bonds with the carboxylic acid group of the acetate ion. The mid-infrared spectrum of potassium acetate intercalated kaolinite showed a comparable but distinctly weaker and broader vibration. Frost et al. (1999) discussed these spectral changes as an indication of the creation of a hydrogen bond between the inner-surface OH-group and the center between the two O atoms in the acetate ion. High and low defect kaolinites are known to exhibit resolved vibrations at 940 and 915 cm^−1^ with an extra vibration at 923 cm^−1^. The two vibrations at 940 and 923 cm^−1^ have been shown to be strongly affected by the inserting acetate anion as they interact with the latter through hydrogen bonding. These two bands were therefore assigned to the OH-deformation modes of the kaolinite interlayer surface OH-groups, which are more accessible to intercalating molecules. Meanwhile, the 915 cm^−1^ band has been attributed to the kaolinite inner OH-group (pointing to the layer towards the tetrahedral sheet) which is shielded from interacting strongly with intercalating molecules. After the kaolinite was intercalated with potassium acetate, two new vibrations were detected around 909 and 897 cm^−1^ for the low defect kaolinite. A third vibration at 877 cm^−1^ was observed for high defect kaolinite and has been assigned to the OH-deformation mode of non-hydrogen-bonded OH-groups. The vibrations at 909 and 897 cm^−1^ have been assigned to the kaolinite inner-surface OH-groups forming hydrogen bonds to water or acetate groups. The intensities of these vibrations are an indication of the degree of disorder created in the kaolinite through intercalation. The intensities of the kaolinite OH-deformation modes were observed to be a function of the original kaolinite defect structure and the method of preparation [31]. These results are consistent with the findings in the OH-stretching region.

The fact that the K 2p3/2 binding energy for both low and high defect kaolinites intercalated with potassium acetate are the same can now be explained by the fact that in both cases the K ion is locked into the ditrigonal hole in the siloxane surface of the tetrahedral sheet exposed to the interlayer space. Clearly, the fact that the carboxylic acid group is involved in forming hydrogen bonds with the hydroxyl groups of the kaolinite octahedral sheet resulting in differences in the mid-infrared and Raman spectra of high and low defect kaolinites is not reflected in the C 1s binding energies of the carboxylic acid group. In contrast, while no differences were observed for the aliphatic group in the vibrational spectra, there was an observable shift in the C 1s binding energy of nearly 0.7 eV. One of the mechanisms thought to be involved in the intercalation was linked to the presence of water molecules in the interlayer space. The binding energy of the third peak at 532.95 eV for the low defect kaolinite intercalated with potassium acetate could be better explained by the presence of water in agreement with vibrational spectroscopy studies instead of separation of the two oxygen atoms in the carboxylic acid group. The presence of water may also have resulted in the shift of the C 1s binding energy of the aliphatic group. Frost, Kristof, Horvath, and Kloprogge [30] observed that upon intercalation several different phases can be formed with different basal spacings as observed by X-ray diffraction (XRD). The first phase is characterized by a basal spacing of 13.9 Å where the acetate ion is hydrogen bonded to a water molecule which in its turn is hydrogen bonded to the OH-groups of the kaolinite octahedral sheet. Slight heating under a nitrogen atmosphere resulted in the development of two different phases with basal spacings of 11.5 Å and 8.9 Å (upon cooling in air the original phase was reformed). The second phase was thought be formed by the carboxyl acid group forming a hydrogen bond to the kaolinite hydroxyl group perpendicular to the interlayer surface, while in the third phase, the carboxylic acid group is thought to have rotated 90° parallel to the interlayer surface. The existence of two orientations of the aliphatic group (perpendicular and parallel to the interlayer surface) may explain the observed difference in the C 1s binding energy for the carboxylic group.

#### 3.1.3. HDTMA Intercalated Montmorillonite

**Figure 4:**
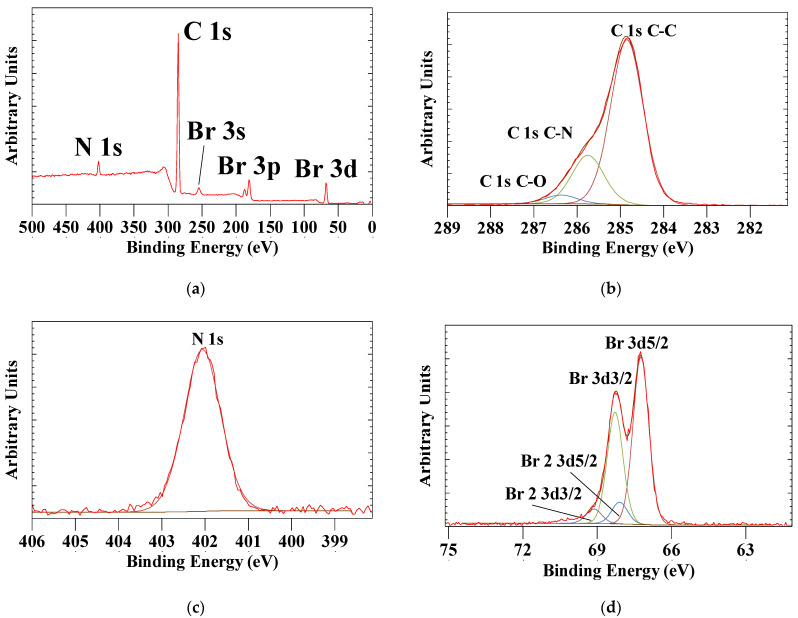
(**a**) XPS survey scan of HDTMABr from 500 to 0 ev, (**b**) high resolution spectrum of C 1s, (**c**) high resolution spectrum of N 1s, and (**d**) high resolution spectrum of Br 3d.

Various organoclays (prepared from smectite group clay minerals, mainly montmorillonite) are used in a wide variety of applications such as nanocomposite precursors, adsorbents for organic pollutants, rheological control agents, electric materials, etc. [18,53,54,55]. Some of these applications can be achieved as a result of the unique properties of montmorillonite arising from an interplay of the layered structure of Mt and hydrophobicity of an intercalated surfactant. Intercalation of a hydrophobic surfactant brings about an expansion of the interlayer space of the clay minerals. Numerous studies have shown that the d-spacings, which is a direct measure of the distance between the two siloxane surfaces in the interlayer space, of the organoclays depends on how long the alkyl chains and how packed the surfactants are within the interlayer space of clay minerals (see, e.g., [56,57,58]). The XPS survey scan is characterized by the peaks associated with N 1s, C 1s and Br 3s, Br 3p, and Br 3d (Figure 4). The high resolution scans show a single N 1s peak with a binding energy of 402.06 and two major C 1s peaks at 284.8 eV associated with the C-C bonds and 285.75 eV associated with the 4 C-N carbon atoms with a ratio of 77 to 23 atom%, which is close to the theoretical ratio of 79 to 21 atom%. The Br 3d shows not one but two different Br- environments. Most of the Br- has a Br 3d5/2 binding energy of 67.19 eV, while a small amount has a slightly higher binding energy of 68.10 eV

**Figure 5:**
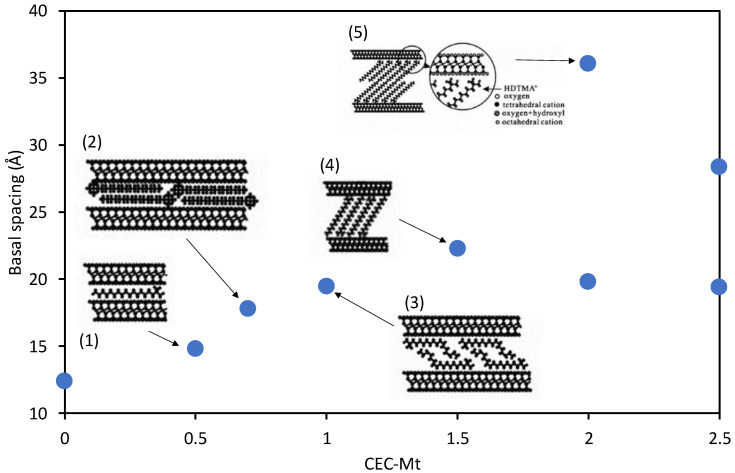
Increase in basal spacing of the HDTMA intercalated montmorillonite as function of the amount of HDTMA relative to the cation exchange capacity of the montmorillonite (CEC), showing the change from lateral monolayer (**1**) to lateral bilayer (**2**), pseudotrilayer (**3**), paraffin monolayer (**4**), and finally to paraffin bilayer (**5**). 0 = Na-montmorillonite. Data used from [34].

An earlier paper by He, Frost, Bostrom, Yuan, Duong, Yang, Xi, and Kloprogge [34] demonstrated that the amount of HDTMA intercalated in montmorillonite resulted in different orientations of the surfactant in the interlayer space. Increases in the XRD basal spacings showed that for the HDTMA intercalated montmorillonite there exist five distinct HDTMA arrangements formed within the montmorillonite interlayer space. These arrangements are lateral monolayer, lateral bilayer, pseudotrilayer, paraffin monolayer, and paraffin bilayer, which are assumed by HDTMA as the CEC increased from 0.5 to 2.5 (Figure 5). The HDTMA arrangement was clearly affected by the packing density of HDTMA within the montmorillonite interlayer space. Transmission and scanning electron microscopy have shown that the intercalated Mt with lower HDTMA packing density primarily exhibited less ordered layer stacking with a number of bent intercalated molecules. In contrast, montmorillonite with higher HDTMA packing densities consisted mainly of a much more ordered layer stacking with less bent intercalated molecules. The variation in interlayer space height from layer to layer was observed in all intercalated Mt, but more obvious variation was observed in the intercalated Mt with lower HDTMA packing density than the one with higher packing density.

**Figure 6:**
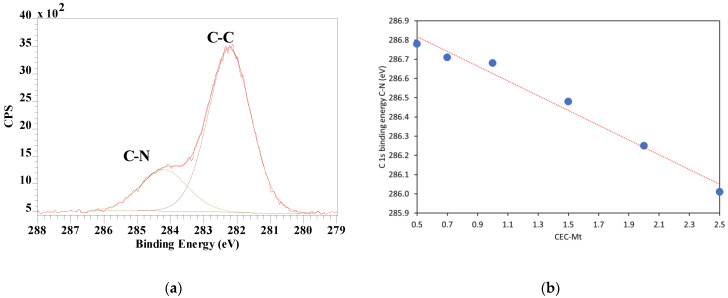
(**a**) Example of a XPS C 1s high resolution scan showing both the C-C and C-N peaks (0.5 CEC-Mt), and (**b**) shift in binding energy of the C-N peak as function of the amount of HDTMA intercalated in Mt.

The C 1s high resolution spectra of HDTMA intercalated montmorillonite (HDTMA-Mt) exhibited a considerable peak broadening with small shifts in binding energy, pointing to more than one type of HDTMA–Mt interaction. The C-C peak remained at 284.8 eV, while the C-N peak showed a shift towards lower binding energies with increasing HDTMA loading (Figure 6). 

The changes in the C 1s high resolution C-N peaks of HDTMA–Mt indicate that the molecular environment surrounding HDTMA has a major influence on the observed binding energy. In all the described arrangements of the HDTMA within the Mt interlayer space, the headgroups (nitrogen) of the alkyl chains are located near the Mt siloxane surface. This is expected as the negatively charged Mt siloxane surface strongly interacts with the positively charged headgroups of the HDTMA. HDTMA–Mt with lower surfactant packing density has the intercalated alkyl chains oriented parallel to the siloxane surfaces in the interlayer space and are well separated. In this type of orientation, repulsions between the HDTMA hydrocarbon groups and the Mt siloxane surface dominate since the interactions of the well separated hydrocarbon chains are extremely weak. An increase in the HDTMA packing density makes the intermolecular interaction among the HDTMA alkyl chains become the main force; the hydrocarbon chain rotates from parallel to the siloxane surface within the Mt interlayer space to an orientation that is at slight angle to the siloxane surface leading to an increase in the basal spacing as shown by XRD [34]. A further increase in the packing density of HDTMA makes the interaction between the HDTMA alkyl chains become stronger causing the arrangement of the alkyl chains to become more orderly as suggested by infrared and Raman spectroscopy [35]. Infrared spectroscopy has shown that both the antisymmetric and symmetric CH_2_ stretching vibrations shifted to lower wavenumbers with increasing HDTMA density in the interlayer space of Mt. This indicated the effect of increased conformation order. The antisymmetric CH_2_ stretching vibration was more susceptible to the conformational ordering. The antisymmetric and symmetric CH_2_ stretching vibrations, on the other hand, were strongly affected by the HDTMA density and orientation. FTIR spectra obtained using pressed KBr disks showed that the splitting of the methylene scissoring and rocking absorption vibrations was strongly dependent on HTDMA density and chain conformation. Two pairs of well resolved vibrations (the first pair at 730 and 720 cm^−1^ and the second pair at 1473 and 1463 cm^−1^) were observed in the HDTMA–Mt with relative higher densities of HDTMA, where the confined HDTMA was present as an essentially all-trans conformation in a paraffin or paraffin bilayer orientation. Similar observations were made using Raman spectroscopy. The local environment of the HDTMA within the intercalated Mt clearly hinge on their loaded amounts with the trimethylammonium heads becoming closer to each other, resulting in the shift of the C 1s binding energy of the C–N bond.

**Figure 7:**
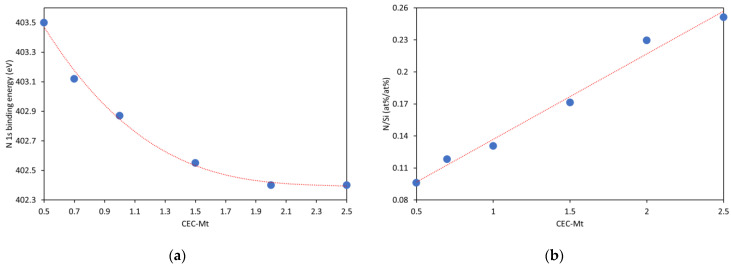
(**a**) Shift in the N 1s binding energy (eV) of HDTMA intercalated in Mt as function of amount of HDTMA intercalated and (**b**) N/Si ratio determined from the atom% of N 1s and Si 2p3/2 high resolution spectra as function of the amount of HDTMA intercalated in Mt.

When high-resolution scans of N 1s were performed for 0.5, 0.7, and 1.0 CEC-Mt, the spectra exhibited a single 1s transition with a slight increase in the binding energy (ca. 1.5 eV) compared with HDMTABr. This indicated that the molecular environment around the intercalated HDTMA is different from that in bulk state HDTMABr. With increasing HDTMA loading, the binding energy shifts to lower values closer to that of bulk HDTMABr (Figure 7a). The atom% ratio of N/Si can be used as a measure of the amount of HDTMA intercalated in Mt (Figure 7b). The N/Si ratio shows a nearly linear increase as would be expected for the intercalation without any excess HDTMA present adsorbed on the external Mt surfaces. In contrast to earlier work by He, Zhou, Frost Ray, Wood Barry, Duong Loc, and Kloprogge [36], no second N 1s peak was observed at high HDTMA loading that they interpreted as externally adsorbed HDTMA on Mt.

**Figure 8:**
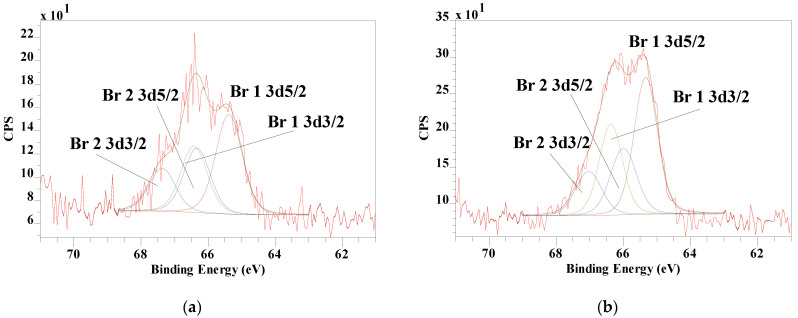
XPS Br 3d high resolution spectra of (**a**) 2.0CEC-Mt and (**b**) 2.5CEC-Mt showing two distinctly different Br^−^ positions labeled Br 1 and Br 2 in the HDTMA-intercalated Mt.

In the survey scans of the HDTMA–Mt, a very weak Br 3d peak was observed in the XPS spectrum of 2.0 CEC-Mt. The intensity of the Br 3d peak distinctly increased in the spectrum of 2.5 CEC-Mt. Figure 8 displays the high-resolution Br 3d high resolution spectra of 2.0 and 2.5 CEC-Mt. The signal is weak due to the fact that the Br is only present in very low concentrations and the release of photoelectrons is a statistical process depending on their release from beneath sample surface. Nevertheless, important details can still be extracted from the spectra. Similar to the HDTMABr Br 3d high resolution spectrum (Br 3d5/2 at 67.19 eV and 68.10 eV), the intercalated HDTMA is also characterized by two different Br 3d5/2 signals at 65.38 eV and a smaller peak at 66.33 eV for 2.0CEC-Mt and at 65.33 eV and 65.99 eV (Figure 8). It is clear from these observations that at high HDTMA intercalation volumes some of the counterion Br^−^ is also included in the Mt interlayer space, although the restraints of the interlayer space result in a significant shift in the binding energies compared with pure HDTMABr. The fact that the peaks are broad with low intensity and poor resolution suggests that the concentration of Br^−^ in the HDTMA–Mt is limited and spatially disordered.

### 3.2. Al- and Ga-Pillared Montmorillonite

Pillared clays (Pilc) form a group of microporous materials prepared through ion exchange of mainly montmorillonite (though other smectites such as saponite, beidellite, and hectorite have also been used) with highly charged metallic complexes followed by a calcination process. Producing suitable Pilc for catalytic applications necessitates comprehensive knowledge of the structure of the precursor clay mineral and the molecule that serves as pillar. Equally important is knowing and understanding where the oxidic pillars are located and what are their size and shape in the final calcined Pilc. These structure-property aspects of Pilc remain poorly understood despite the vast amount of research conducted over the past several decades in the field of Pilc structures. In this section, XPS is used to probe the pillaring of montmorillonites (Mt) with Al and Ga polyoxocations to gain more information on the pillaring process and the resulting Pilc. These are chosen as the model systems since pillaring of Mt by polyoxocations has been researched for more than 5 decades. Al_13_-montmorillonites are the most created Pilc. This is largely due to the large basal d-spacing created by Al polyoxocation in the Pilc as well as its relative stability at high temperatures even up to temperatures where the clay layers start to break down due to dehydroxylation (loss of the octahedral OH-groups). This high temperature stability is also exhibited by the very similar Al_12_Ga-Pilc. The Al_13_, Al_12_Ga, and Ga_13_ have equivalent structural form—all consisting of a Keggin structure with the central Al^IV^O_4_ or Ga^IV^O_4_ tetrahedron caged by twelve Al^VI^ octahedra with H_2_O and OH-groups producing similar basal d-spacings in the final Pilc [10,59,60,61,62,63,64,65].

#### 3.2.1. Al_13_ Pillared Montmorillonite

The XPS spectroscopic results for montmorillonite SWy-2 used in the pillaring studies was described by Kloprogge and Wood [47]. Pillaring with Al_13_ results in a strong increase in the amount of total Al in the Pilc structure with the Si/Al ratio decreasing from 2.34 for the starting Mt to 0.96 for the Pilc. Based on these ratios, it is possible to calculate that of the total Al in the Pilc about 40% is due to Al in the octahedral sheet of the Mt, while 60% is due to the pillar structure in the Mt interlayer space. Given a total of 4 Si atoms per unit cell for Mt it is possible to calculate the theoretical number of pillars assuming that 13 Al atoms are present in each pillar. Based on the Si and Al atom% for the Mt and Pilc, an average of one pillar per five unit cells was present in the Pilc. The Si 2p3/2 peak in the Si 2p high resolution scan shifted from 102.51 eV for Mt to 99.68 eV for the Pilc. This shift can be explained by the fact that upon calcination of the intercalated Al_13_-Mt new oxide bonds are formed between the Al in the pillar structure and the siloxane surface through inversion of some Al tetrahedra into the Mt interlayer space [66,67].

**Figure 9:**
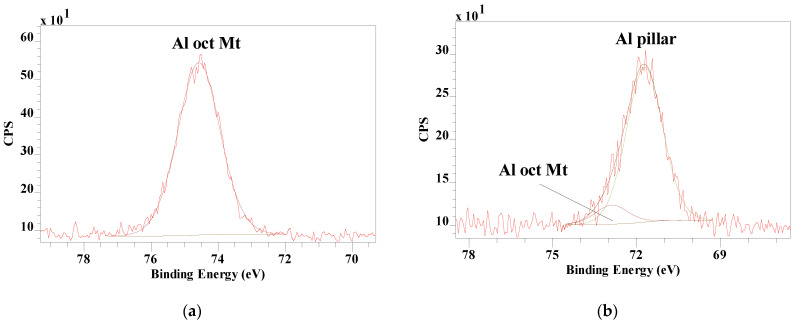
XPS Al 2p high resolution spectra of (**a**) Mt and (**b**) Al_13_-Pilc after calcination.

The single Al 2p at 74.58 eV for Mt split into a strong peak at 71.78 eV and a smaller peak at 72.89 eV (Figure 9). The smaller peak is probably associated with the octahedral Al in the Mt layer, while the strong peak can be assigned to the Al in the pillar. Even though the Al in the octahedral sheet is relatively shielded from the newly formed structure through the oxygen bond to the tetrahedral sheets, it still shows an influence of the structural change on the binding energy. It has been suggested that the structure of the pillar resembles that of corundum (Al_2_O_3_) but the Al 2p binding energy for corundum is 74.1 eV [20], which is significantly higher than observed here for the pillar. 

Kloprogge, Frost, and Fry [41] showed that the infrared spectrum of the OH-stretching region of montmorillonite is depicted by two vibrations at 3628 and 3363 cm^−1^ attributed to the octahedral sheet’s M–OH and interlayer H_2_O OH-stretching modes. New vibrations appear at 3682 and 3538 cm^−1^ and the interlayer H_2_O OH band moved to 3200 cm^−1^ when Mt is intercalated with Al_13_. The new vibrations are attributed to the OH-stretching modes of the Al–OH and Al–H_2_O groups of the Al_13_ complex. The interlayer H_2_O band strongly decreased in intensity as a result of the intercalation with Al_13_. Calcination caused the loss of any residual interlayer water. The transformation of the Al_13_ Keggin structure caused the disappearance of the Al–OH stretching vibration at 400–500 °C. Between 400 and 500 °C, the OH-stretching vibration of the Al–H_2_O group was replaced by two new vibrations at 3574 and 3505 cm^−1^. This is indicative of rearrangements within the Al_13_ structure. These vibrations were still visible at 800 °C albeit with a significant decrease in intensity. Nonetheless, this indicated that a few structural OH-groups remained in the newly formed pillar structure, contrary to the common belief that these were completely converted into aluminum oxide. These bands were thought to possibly represent OH bridges between neighboring partially dehydroxylated Al_13_ complexes. The M–OH vibration was observed over the entire temperature range from room temperature up to 800 °C without position shifts, though with diminishing intensity [35]. These infrared emission spectroscopic results fit with the observed Al 2p binding energy for Al in the pillar, showing that the structure is not the same as that observed in corundum.

#### 3.2.2. Ga_13_ Pillared Montmorillonite

The work of Duong et al. [39] on the distribution of Ga in Ga_13_-pillared montmorillonites showed that the XRD profile upon intercalation of the Ga_13_ exchanged Mt from Miles, Queensland had a basal spacing of about 19.9 Å compared with 14 Å for the starting Mt, which slightly decreased upon calcination to 17.9 Å similar to Al_13_-Pilc. Earlier analytical work using energy dispersive X-ray analysis (EDX) has shown that that mean atom ratio of Si/Ga was about 4.25 [39]. Compared with Mt SWy-2, this indicates a three times higher pillar density in the Miles Al_13_- and Ga_13_-Pilc, which is probably the result of a higher layer charge in the Miles Mt compared with SWy-2.

**Figure 10:**
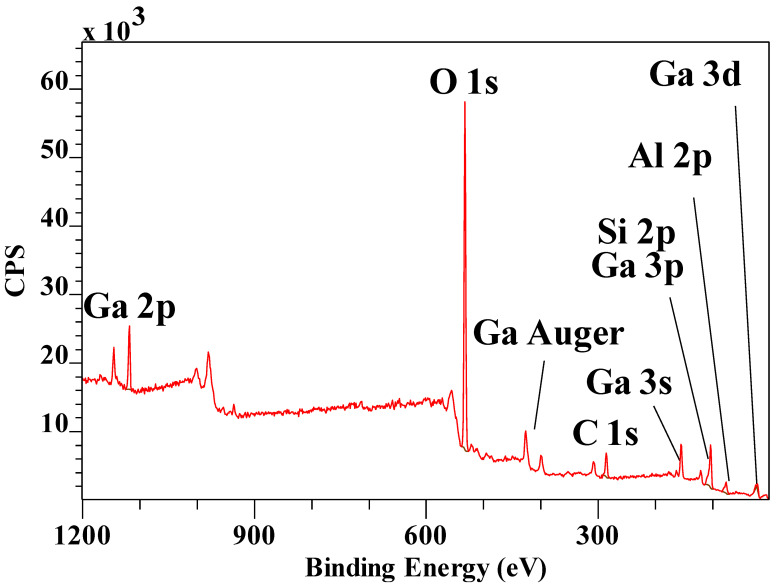
XPS survey scan of Ga_13_ pillared montmorillonite.

**Figure 11:**
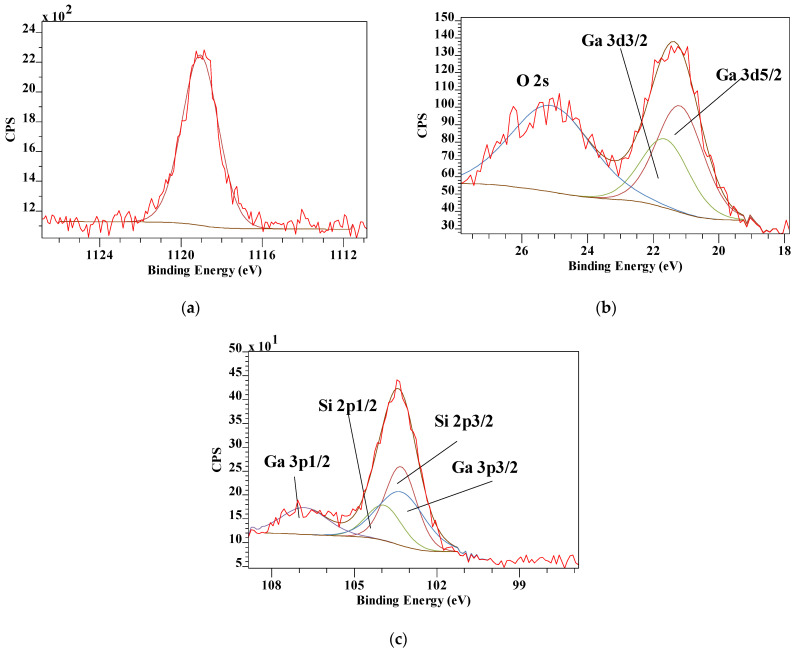
XPS high resolution scans of (**a**) Ga 2p3/2. (**b**) Ga 3d showing overlap with O 1s and (**c**) Ga 3p showing overlap with Si 2p.

Figure 10 shows the XPS survey scan of the Ga_13_ pillared montmorillonite. The Ga peaks are clearly identifiable, though there is significant interference with some other elements as the high resolution scans show. Ga 3d overlaps with O 2s, while Ga 3p overlaps with Si 2p (Figure 11). The ratio of Si/Ga obtained from the XPS scans is significantly lower at 1.61 compared with the earlier reported EDX value of 4.25, indicating nearly three times the amount of Ga. As shown above, there is significant overlap with several of the Ga peaks with Si and O in the XPS spectra. Similarly, there is interference in the EDX patterns as well with Ga overlapping with, for example, Mg, introducing uncertainty in that value as well. Similar to the Al_13_-Pilc, the Si 2p3/2 has shifted to lower binding energy by about 2 eV, while the corresponding Al 2p has shifted to lower binding energy by about 1 eV, both of which are significantly smaller than for the Al_13_-Pilc. This may be related to the fact that a different starting Mt was used with different amounts of substitutions resulting in different layer charges as indicated by the differences in the Si/Al ratio for Al_13_-Pilc in the previous section compared with the Si/Ga ratio here. This is supported by the fact that the cation exchange capacity (CEC) for the Miles Mt is significantly higher than that of SWy-2 [39].

The Ga 2p3/2, 3p, and 3d high resolution spectra all show a single peak indicating that after calcination the Ga environment of all Ga atoms is very similar. Compared with gallium-substituted boehmite, (Al,Ga)OOH, with all Ga in octahedral coordination where the Ga 2p3/2 occurred at 1117.8 eV [21], the Ga in the Ga_13_-Pilc is observed at a higher binding energy of 1119.1 eV. In the previous section it was discussed how the Al_13_ pillars after calcination still contained some hydroxyl groups, which is probably also the case in the Ga_13_ pillars here, but not enough to shift the Ga 2p3/2 to a binding energy similar to that in Ga-substituted boehmite. Montarges et al. [68] used Ga K-edge X-ray adsorption to study Ga_13_-Pilc to probe the local environment of Ga atoms Ga_13_-Mt. Their analysis of the first nearest neighbors (oxygen atoms neighbors) revealed a reduction in the average Ga-OH distances after calcination. This was interpreted as due to the dehydroxylation of the polycation pillars in Ga_13_-Pilc. For the second nearest gallium atom neighbors, the Fourier transforms pointed to the initial existence of the central Ga^IV^ of the Ga_13_ polycations. Their signal contribution, however, disappeared upon calcination in accordance with the observed XPS results in this study. The ^71^Ga Nuclear Magnetic Resonance (NMR) spectra of partly hydrolyzed Ga^3+^ solutions exhibited a peak at 176 ppm, assigned to the central Ga^IV^ of the Ga_13_ polymer [40,69,70]. The ^71^Ga solid state Magic Angle Spinning (MAS) NMR spectra of Ga_13_-Pilc at 25 °C and calcined 150 °C showed a tetrahedral peak at 172.6 ppm and an octahedral signal at 30.1 ppm. Upon calcination at 300 °C, these signals were no longer observed. Instead, a very broad signal at ~0 ppm was observed indicating that the central Ga^IV^ in the original Ga_13_ Keggin structure was transformed to Ga^VI^ in the pillar structure, further supporting the XPS results [40].

#### 3.2.3. Al_12_Ga Pillared Montmorillonite

**Figure 12:**
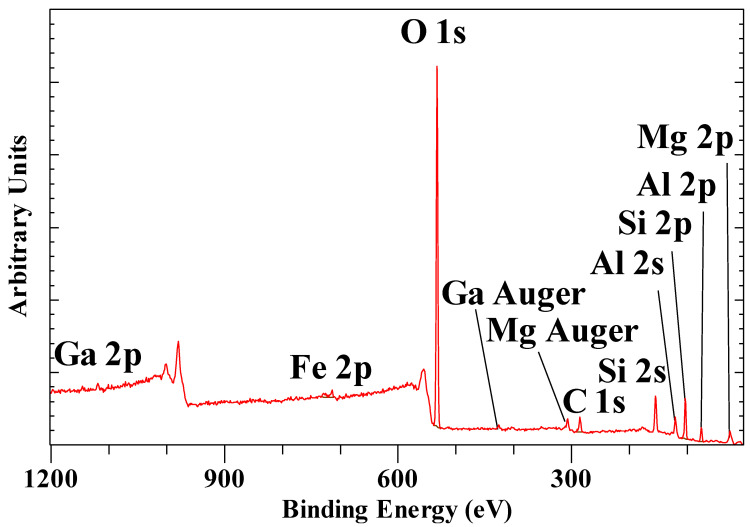
XPS survey scan of Al_12_Ga pillared montmorillonite.

The XPS survey scan of Al_12_Ga-Pilc is shown in Figure 12. The Ga 2p is barely visible compared with the intensity observed for Ga_13_-Pilc in Figure 10. The concentration of Ga as determined from the XPS scans compared with the concentration of Ga in the Ga_13_-Pilc is much lower as expected. The atom ratio of Ga in Al_12_Ga to Ga_13_ is 1/11.9, close to the expected ratio of 1/13. The Si 2p3/2 has shifted to a slightly lower binding energy by about 0.4 eV, which is just above the instrumental error, while the Al 2p has not shifted in comparison with Ga_13_-Pilc. The Ga 2p3/2 is much weaker and has a binding energy of 1119.7 eV, which is slightly higher that observed for Ga_13_-Pilc in the previous section at 1119.1 eV. This small shift in binding energy may be because of the fact that the central Ga^IV^ atom fits significantly better in the Al_12_Ga Keggin structure compared with Al_13_ and Ga_13_ [71]. Overall, these results agree with those discussed in the previous sections on Al_13_ and Ga_13_ pillared montmorillonite.

### 3.3. Hexacyanoferrate Intercalated Hydrotalcite

Layered double hydroxides (LDH), of which hydrotalcite is a member, are fundamentally anionic clays [1,72]. The hydrotalcite structure can be taken as a derivative of brucite, (Mg(OH)_2_), wherein a few Mg^2+^ are substituted by a trivalent metal such as Fe^3+^ or Al^3+^ resulting in a net positive charge of the hydroxide layer. The layer charge is offset by interlayer anions or by negatively charged complexes, opposite to the normal silicate clay minerals where the negative layer charge is compensated by positive interlayer cations (hence the name anionic clays). The range of compositions that are feasible for LDH of the type [$M_{1-x}^{2+}$ $M_{x}^{3+}$ (OH)_2_][A^n−^]_x/n_.yH_2_O is large. Here, M^2+^ and M^3+^ are octahedrally-coordinated di and trivalent cations within the hydroxide layers while A^n−^ is the exchangeable interlayer anion. The values of x are typically between 0.17 and 0.33. 

**Figure 13:**
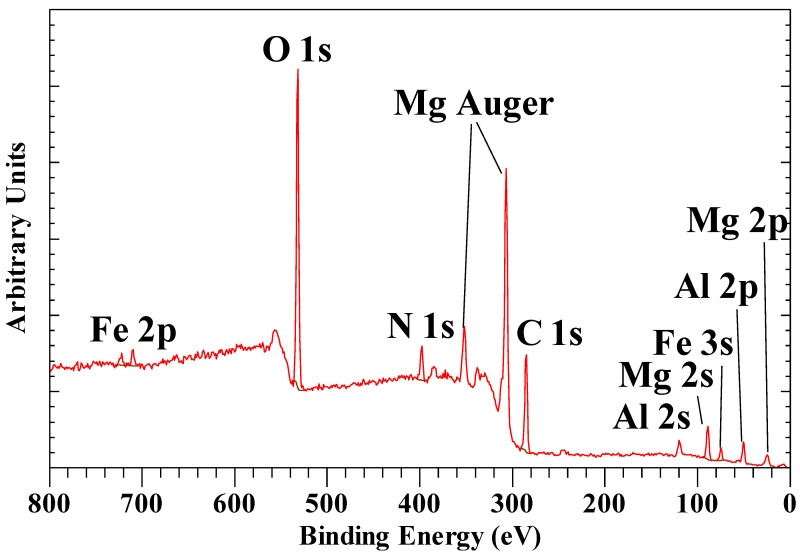
XPS survey scan from 800 to 0 eV of [Fe^3+^(CN)_6_]^3−^ intercalated hydrotalcite.

Figure 13 shows the XPS survey scan of hexacyanoferrate(III) intercalated hydrotalcite. The hydrotalcite structure is characterized by a single Mg 2p peak at 50.03 eV, a single Al 2p peak at 74.51 eV, and finally a single O 1s peak at 531.78 eV. For the hexacyanoferrate(II) intercalated hydrotalcite, these peaks are observed with the same binding energies. The Mg/Al ratios are 2.17 and 2.21, respectively, which corresponds to x = ~ 0.3 in the LDH formula provided in the previous paragraph. Kloprogge and Wood [73] published the full XPS spectrum of hydrotalcite with Mg 2p at 50.1 eV, Al 2p at 74.5, and O 1s at 531.6 eV for OH in the layers, which is in perfect agreement with the data shown here for the intercalated hydrotalcite. Peng, et al. [74] reported similar results for hydrotalcite with Mg 2p at 50.29 eV, Al 2p at 74.31 eV, while Lucrédio, et al. [75] observed Al 2p for calcined Co/Al/Mg LDH around 71.1 eV and O 1s around 530 eV. They did not report the Mg 2p but the Mg 2s instead at 87.2- to 88.3 eV, which compares well with the value of 88.73 eV observed here. The observed Al 2p peak at 74.51 eV agrees well with the values observed for various Al oxy(hydroxides) such as gibbsite (74.4 eV), bayerite (75.0 eV), boehmite (73.9 eV), and pseudoboehmite (74.3 eV) [20]. The Mg 2p for Mg(OH)_2_ has been reported at 49.3 eV, which is slightly lower than for hydrotalcite due to the partial substitution of Mg^2+^ by Al^3+^ [76]. 

**Figure 14:**
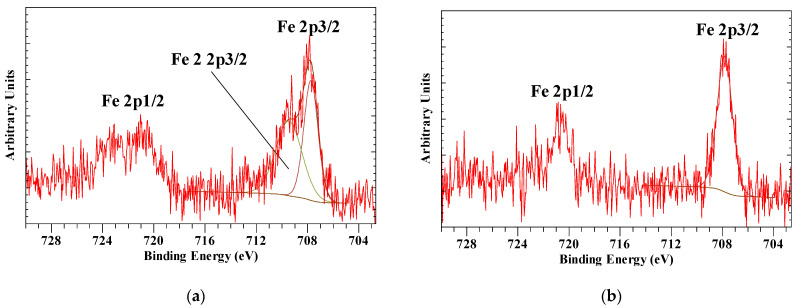
XPS high resolution scans of (**a**) hexacyanoferrate(III) intercalated hydrotalcite and (**b**) hexacyanoferrate(II) intercalated hydrotalcite.

The intercalating anion is characterized by a single N 1s peak at 397.43 eV for the hexacyanoferrate(III) anion. For the hexacyanoferrate(II) anion the N 1s peak was found at 397.11 eV. There are significant differences in the Fe 2p3/2 peaks with the hexacyanoferrate(III) showing the main peak at 707.74 eV and a second peak at 709.46 eV, while hexacyanoferrate(II) shows a single peak at 707.81 eV (Figure 14). Holgado, Rives, Sanromán, and Malet [38] showed based on Fe–K XANES analysis that partial reduction of the Fe(III) in the hexacyanoferrate intercalated hydrotalcite had occurred. In addition, Idemura et al. [77] showed the reduction of iron in hexacyanoferrate(III) complexes by Mössbauer spectroscopy. Yamashita and Hayes [26] observed that the Fe 2p3/2 for Fe(II) has a binding energy of about 1 eV lower than Fe(III) in their oxides. Similar results have been reported for thin iron oxide films [19] and iron sodium glasses [78]. Based on this, it can be concluded that the Fe 2p3/2 peak at 707.74 eV is due to Fe(II) due to partial reduction in the initial Fe(III) observed at 709.46 eV.

## 4. Conclusions

XPS was used to probe the local environments of intercalating or pillaring organic and inorganic molecules within the interlayer surface of different types of clay minerals and a layered double hydroxide (LDH or “anionic clay”). The preparation of these intercalated systems is dependent on the desired applications by optimizing and enhancing the properties of the resulting products. Understanding the size, location, and structural changes of the pillaring/intercalating compound within the clay mineral interlayer space is important as it would impact the properties of the final modified clay. Raman and infrared spectroscopy together with XRD are the commonly used analytical techniques to these intercalated systems. Yet, there are additional insights that can be provided by XPS that cannot be obtained using these techniques as shown in this study. XPS of urea-intercalated kaolinite exhibited a significant shift of the Si 2p3/2 binding energy by 2 eV revealing the influence of NH_2_ on the siloxane group, which is contrary to observations from Raman and IR that urea only interacted with the -OH groups of the octahedral sheet where aluminum is located. XPS survey of K-acetate intercalated high- and low-defect kaolinite showed that the K 2p3/2 peak was unaffected by the kaolinite defect structure and interpreted as having the K ion in both cases locked in the ditrigonal hole of the siloxane surface of the tetrahedral sheet exposed to the interlayer space. There were observed differences in the binding energies of the aliphatic group and the O 1s of the kaolinite structure of the two types of kaolinite where the low defect kaolinite is said to promote better ordering of K-acetate in the interlayer space to warrant the observation of the two oxygen atoms in carboxylic acid separately. In the intercalation of montmorillonite with HDTMA, the peak broadening and small shift in binding energies corroborates information provided by other techniques on the different types of orientation adopted by HDTMA within the interlayer space of montmorillonite with varying CEC values. XPS of Ga_13_- and Al_13_- and Al_12_Ga-pillared montmorillonite provided insights into the coordination of the pillaring compound within the Mt layer and the transformations they underwent upon calcination as well as the size of the polycation that fits better in the Keggin structure of the pillar. Lastly, for hexacyanoferrate intercalated hydrotalcite, XPS revealed changes in the oxidation state of the iron in the intercalating molecule. It is clear from these examples that shifts in binding energies and presence of multiple peaks in XPS can provide information on the different environments surrounding a particular atom. Each element produces a specific XPS signal which can be correlated to its electronic configuration which can also be quantified in relation to its sampling volume. Size, coordination, and structural transformations of the intercalating/pillaring compounds and the changes in their oxidation states provide useful information that can be used to design intercalated systems with a specific type of interaction to enhance the desired properties of these modified systems. When coupled with FTIR, RAMAN, and XRD, the overall information that can be generated for these modified systems provides a stronger handle on the synthesis and quality of new materials produced.
